# Efficient and robust differentiation of endothelial cells from human induced pluripotent stem cells via lineage control with VEGF and cyclic AMP

**DOI:** 10.1371/journal.pone.0173271

**Published:** 2017-03-13

**Authors:** Takeshi Ikuno, Hidetoshi Masumoto, Kohei Yamamizu, Miki Yoshioka, Kenji Minakata, Tadashi Ikeda, Ryuzo Sakata, Jun K. Yamashita

**Affiliations:** 1 Department of Cell Growth and Differentiation, Center for iPS Cell Research and Application, Kyoto University, Kyoto, Japan; 2 Department of Cardiovascular Surgery, Kyoto University Graduate School of Medicine, Kyoto, Japan; University of Kansas Medical Center, UNITED STATES

## Abstract

Blood vessels are essential components for many tissues and organs. Thus, efficient induction of endothelial cells (ECs) from human pluripotent stem cells is a key method for generating higher tissue structures entirely from stem cells. We previously established an EC differentiation system with mouse pluripotent stem cells to show that vascular endothelial growth factor (VEGF) is essential to induce ECs and that cyclic adenosine monophosphate (cAMP) synergistically enhances VEGF effects. Here we report an efficient and robust EC differentiation method from human pluripotent stem cell lines based on a 2D monolayer, serum-free culture. We controlled the direction of differentiation from mesoderm to ECs using stage-specific stimulation with VEGF and cAMP combined with the elimination of non-responder cells at early EC stage. This “stimulation-elimination” method robustly achieved very high efficiency (>99%) and yield (>10 ECs from 1 hiPSC input) of EC differentiation, with no purification of ECs after differentiation. We believe this method will be a valuable technological basis broadly for regenerative medicine and 3D tissue engineering.

## Introduction

Blood vessels play essential roles in the generation of higher tissue structures, especially large tissue and organ structures. The importance of endothelial cells (ECs) has already been shown in the formation of various organs such as heart[1–3], liver[4–7], kidney[8], bone[9], and skin among many others[10–13]. Thus, efficient EC preparation methods that provide scalable and stable supply are necessary for three-dimensional (3D) tissue engineering and organ regeneration. Human pluripotent stem cells are one of the most suitable sources for such purpose.

Previously, using mouse embryonic stem cells (ESCs), we established a method for systematic induction of cardiovascular cells from vascular endothelial growth factor (VEGF) receptor-2 (VEGFR2)-positive mesoderm cells as cardiovascular progenitors[14,15]. VEGF/VEGFR2 signaling is essential for inducing EC differentiation from VEGFR2-positive mesoderm cells. Furthermore, we also found that cyclic adenosine monophosphate (cAMP) signaling potently enhances EC differentiation[16,17] and that activation of a major downstream molecule of cAMP, protein kinase A (PKA), increased the expression of VEGFR2 and another VEGF receptor, neuropilin1, which together form a specific receptor for the VEGF-A_165_ isoform. The binding of VEGF-A_165_ to VEGFR2 and neuropilin1 is reported to enhance VEGFR signaling by approximately a factor of ten. Coincidently, PKA activation increased the sensitivity of VEGFR2^+^ progenitors to VEGF, which increased the appearance of ECs also by a factor of ten[17]. PKA is also directly involved in the EC commitment process. Etv2/ER71, an ETS transcription factor, plays an indispensable role in EC and hematopoietic lineage commitment from early mesoderm[18,19]. We previously showed that PKA-activated CREB (cyclic AMP-responsive element (CRE) binding protein) bound to CRE on the Etv2/ER71 promoter region and directly induced Etv2/ER71 expression[20]. In that same report, we also observed that PKA activation during ESC differentiation triggered EC differentiation and induced early commitment to EC lineage. In addition, we reported that Notch and β-catenin signaling are simultaneously activated in the downstream of cAMP and protein complex formation with Notch intracellular domain and β-catenin induced a set of arterial EC gene expressions resulted in arterial EC differentiation[21]. These results indicate that VEGF is critical for EC differentiation and growth, while cAMP is critical for EC commitment and specification.

With regards to human induced pluripotent stem cell (hiPSC) differentiation, we previously reported an efficient cardiomyocyte (CM) differentiation method based on a 2D monolayer, serum-free condition[22], that was modified from a directed differentiation protocol from human ESCs[23]. In our method, we first induce mesoderm cells with Activin-A, bone morphogenic protein 4 (BMP4), and basic fibroblast growth factor (bFGF), and then induced CM commitment with a wnt inhibitor, Dickkopf-related protein 1 (DKK1). Inferring from our mouse ESC results, we anticipated that fate control of mesoderm stage cells to EC lineage should provide an efficient source of ECs. Therefore, in the present study, we investigated an EC differentiation method from hiPSCs that combined differentiation stage-specific supplementation of VEGF and cAMP. We further demonstrated that purification of EC-committed cells at peri-EC stage, which can eliminate non-responder cells to EC differentiation ques, achieved highly pure and efficient EC differentiation.

## Materials and methods

### hiPSC culture and differentiation

**Fig 1:**
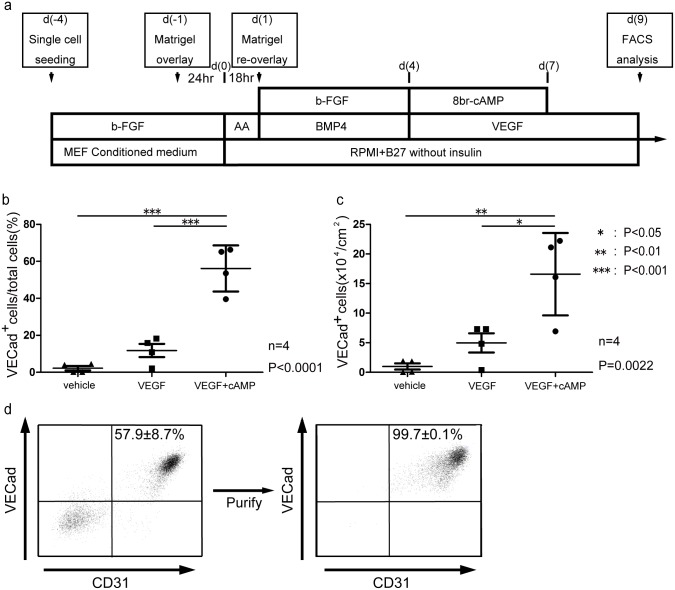
Efficient induction of endothelial cells from human pluripotent stem cells with supplementation of VEGF and cAMP. (a) Schematic representation of the stimulation method. (b) Ratio of VE-Cadherin positive cell per total cells at differentiation day 9 by flow cytometory in stimulation method group (VEGF+cAMP), VEGF administration groups (VEGF) and no administration groups (vehicle). (c) Mean yield of endothelial cells per 1cm^2^ in three groups. (d) Distinct expression pattern of VE-cadherin in stimulation method at differentiation day 9 (left panel) and post-VE-Cadherin purification (right panel).

Three hiPSC lines, 201B6, 201B7 and 836B3, were used. 201B6 and 201B7 were established by retroviral transduction using 4 factors: Oct3/4, Sox2, Klf4, and c-Myc[24]; 836B3 was established by episomal plasmid vector transfection using 6 factors: Oct3/4, Sox2, Klf4, L-Myc, LIN28 and Glis1[25]. 201B6 was used in all experiments except for the confirmation of multiple cell lines. hiPSCs were maintained in a feeder-free condition with conditioned medium of mouse embryonic fibroblasts (MEF-CM) supplemented with 4 ng/mL human basic fibroblast growth factor (bFGF; WAKO, Tokyo, Japan) on thin-coated Matrigel (Growth factor reduced; 1:60 dilution; Corning, Corning, NY). MEF cells were plated at approximately 55,000 cells/cm^2^ in MEF medium (Dulbecco’s modified Eagle’s medium (DMEM) (Nakalai Tesque, Kyoto, Japan) containing 10% fetal calf serum (FCS) (Thermo Fisher Scientific, Waltham, MA), 2 mM L-glutamine, 1% nonessential amino acids (NEAA) (Thermo Fisher Scientific)) followed by treatment with mitomycin-C (MMC) (WAKO) for 2.5 hours. One day after plating, culture medium was changed to ES medium (80% Knockout DMEM (Thermo Fisher Scientific), 20% KSR (Thermo Fisher Scientific), 1 mM L-glutamine, 0.1 mM β-mercaptoethanol (Thermo Fisher Scientific), 1% NEAA, and 4 ng/mL hbFGF (WAKO); 0.5 mL/cm^2^), and MEF-CM was collected daily for 1 week. hiPSCs were passaged every 4–6 days as small clumps using CTK solution (0.1% Collagenase IV, 0.25% Trypsin (Thermo Fisher Scientific), 20% Knockout serum replacement (KSR), and 1 mM CaCl_2_ in phosphate buffered saline (PBS)). The endothelial differentiation process is summarized in Fig 1a. In detail, cells were dissociated using Versene (Thermo Fisher Scientific) for 3–5 min in an incubator at 37°C and seeded onto Matrigel thin-coated plates at 60,000 to 87,500 cells/cm^2^ in 1 mL of MEF-CM with 4 ng/mL of bFGF four days before EC induction. Matrigel solution (1:60 dilution) was added to the cells 24 hours before EC induction. To induce endothelial differentiation, we replaced MEF-CM with RPMI+B27 medium (RPMI1640 (Thermo Fisher Scientific), 2 mM L-glutamine, 1xB27 supplement without insulin (Thermo Fisher Scientific)) supplemented with 125 ng/mL of Activin A (R&D Systems, Minneapolis, MN) and treated for 18 hours and then changed the culture medium to 10 ng/ mL human bone morphogenetic protein 4 (BMP4; R&D), 10 ng/mL bFGF and Matrigel (1:60 dilution) and treated for 3 days. The culture medium was subsequently replaced with RPMI+B27 supplemented with 1 mM 8bromo-cAMP (Nakalai Tesque) and 100 ng/mL VEGF (WAKO) at differentiation day 4 (d(4)) and cultured for 3 days. The culture medium was refreshed on d6. On d7, the culture medium was changed to RPMI+B27 with the same dose of VEGF without 8bromo-cAMP and refreshed every 2 days.

### HUVEC and HUAEC culture

Human Umbilical Vein Endothelial Cells (HUVECs) were from Lonza (Basel, Switzerland) and maintained in EGM-2 medium (Lonza). Cells were isolated using 0.25%trypsin (Thermo Fisher Scientific), then cultured on none-coated culture dish at 2,500–5,000 cells/cm^2^. Human Umbilical Artery Endothelial Cells (HUAECs) (Promo Cell, Heidelberg, Germany) were cultured in Endothelial Cell Growth Medium (Promo Cell). DetachKit (Promo Cell) were used for digestion, and then the HUAECs were re-cultured at 4,000–5,000 cells/cm^2^. Cells were passaged every 2-3days. At passage 3–5 cells were used for experiments.

### Flow cytometry

On d(9), we dissociated the cells using Accumax (Innovative Cell Technologies, San Diego, CA) and stained them with the cell surface markers listed in S1 Table. For cell surface markers, staining was carried out in PBS with 5% FCS. To eliminate dead cells, cells were stained with 4’,6-diamidino-2- phenylindole (DAPI) for surface marker staining or with the LIVE/ DEAD fixable Aqua Dead Cell Staining Kit (Thermo Fisher Scientific) for intracellular staining. For intracellular proteins, staining was carried out on cells fixed with 4% paraformaldehyde (PFA) in PBS. Cells were stained with the anti-cardiac isoform of Troponin T (TNNT2) (clone 13211, Thermo Fisher scientific) labeled with Alexa-488 using Zenon technology (Thermo Fisher Scientific). The staining was performed in PBS with 5% FCS and 0.75% Saponin (Sigma-Aldrich, St. Louis, MO). Stained cells were analyzed on an AriaII flow cytometer (Becton Dickinson, Franklin Lakes, NJ). After the selection of FSC/SSC gate, we additionally eliminated the doublets by SSC-W/SSC-H gate and FSC-W/FSC-H gate. Data were collected from at least 10,000 events.

### Evaluation of Vascular Endothelial (VE)-cadherin^+^ ECs at d(9)

At d(9), cells were dissociated using Accumax and stained with DAPI and VE-Cadherin. Staining was carried out in PBS with 5% FCS. Stained cells were examined and purified on an AriaII flow cytometer. Viable cells were counted with the trypan blue-exclusion test. The yield of ECs was calculated by multiplying the viable cell count with the percentage of ECs.

### Purification and re-culture of VEGF Receptor-2 (VEGFR2)^+^ cells at d(6)

At d(6), cells were dissociated using Accumax and stained with DAPI and VEGFR2. VEGFR2-positive cells were purified on an AriaII flow cytometer and then recultured at 10,000 VEGFR2^+^ cells/cm^2^ in RPMI+B27 medium with 1 mM 8bromo-cAMP, 100 ng/mL VEGF and 10 μmol/L of a Rho-associated coiled-coil forming kinase inhibitor (Y-27632; CalBiochem) on thin-coated Matrigel dishes. The culture medium was replaced to that supplemented with VEGF alone on d(7). On d(9), viable cells was counted by the trypan blue-exclusion test. The purity of VE-cadherin and CD31 double-positive ECs was analyzed with a flow cytometer.

### Pure hiPSC-derived EC culture

hiPSC-derived ECs with more than 99% purity were obtained by the stimulation method or stimulation-elimination method (see Results). hiPSC-derived ECs were plated on 1% gelatin-coated dishes in human endothelial-serum free medium (SFM; Thermo Fisher Scientific) with 20 ng/mL bFGF, 10 ng/mL EGF, and 10 μg/mL human plasma fibronectin at 10,000 cells/cm^2^. ECs grew at 80–90% confluency within 5–7 days.

### Immunofluorescent imaging

Cells were fixed with 4% PFA and stained with antibodies shown in S1 Table. Nuclei were visualized with DAPI (Thermo Fisher Scientific). Stained cells were photographed with an all-in-one fluorescent microscopic system, Biorevo BZ-9000 (Keyence, Osaka, Japan).

### Tube formation assay

Tube formation assay was performed as described previously[26]. Briefly, differentiated ECs (7 x 10^4^) at d13 were plated on a 24-well plate (Thermo Fisher Scientific) coated with 300 μl Matrigel Basement Membrane Matrix GFR (BD Biosciences), and cultured for 24 hrs.

### LDL uptake

Assay of LDL uptake was performed as described previously[27]. Briefly, LDL uptake by cells was assessed by fluorescent microscopy after incubation of the differentiated ECs with 10 μg/ml acetylated LDL labeled with 1,1’-dioctadecyl-3,3,3’,3’-tetramethylindo-carbocyanine perchlorate (DiI-Ac-LDL) (Biomedical Technologies, Stoughton, MA) for 4 h at 37°C.

### Arterial and venous EC culture

VEGFR2-positive cells in the stimulation-elimination method at d(6) were separated into four groups. i) basal group: same as the stimulation-elimination method (VEGF from d(4) to d(9) and 8bromo-cAMP from d(4) to d(6)), ii) extended cAMP group: extended 1mM 8bromo-cAMP from d(7) to d(14), iii) Ang1 group: additional angiopoietin 1 from d(7) to d(9), and iv) VEGF alone group: VEGF alone from d(4) to d(9). EC phenotypes were examined by FACS at d(6), d(9), and d(14).

### Quantitative reverse-transcription Polymerase Chain Reaction (qPCR)

Total RNA was extracted from purified ECs using RNeasy (QIAGEN, Hilden, Germany) according to the manufacturer’s instructions. GAPDH was used to normalize gene expressions. Quantitative PCR was performed using Power SYBR Green PCR Master Mix (Thermo Fisher Scientific) on a StepOnePlus system (Thermo Fisher Scientific) with Delta Delta Ct method. Forward and reverse primer sequences are shown in S2 Table.

### Statistical analysis

At least three independent experiments were performed. Statistical analysis of the data was performed with ANOVA. p < 0.05 was considered significant. Values are reported as mean ± SD.

## Results

### Optimization of VEGF and cAMP supplementation

Previously, we established a monolayer high-density culture-based CM differentiation protocol from hiPSCs[22,23]. In the present work, we attempted to induce ECs from hiPSCs by directing the fate of mesoderm-stage cells toward ECs mainly by VEGF and cAMP activation. Briefly, mesoderm-stage cell induction process is as follows: undifferentiated hiPSCs maintained on feeder-free condition were collected as single cells, and seeded on Matrigel-coated multi-well plates 4 days prior to the initiation of differentiation (d(-4)). Matrigel overlay was conducted when the culture became fully confluent (d(-1)) for 24 hours, then we initiated the differentiation culture (d(0)). Mesoderm cells were induced with the addition of Activin-A (100 ng/mL) from d(0) to d(1) followed by BMP4 (10 ng/mL) and bFGF (10 ng/mL) from d(1–5).

Then, various concentrations of VEGF and a cAMP analogue, 8-bromo-cAMP, were supplemented at time points around the possible mesoderm stage (d(5)), and the efficiency of vascular endothelial (VE)-cadherin-positive EC induction on d(9) was evaluated with flow cytometry. First, we examined the effects of VEGF. The addition of VEGF on d(5–9) dramatically induced EC appearance compared with no VEGF. VEGF (100 ng/mL) stimulated efficient VE-cadherin^+^ EC appearance whereas VEGF at 200 ng/mL showed no apparent difference with VEGF 100 ng/mL (S1a Fig). The addition of VEGF on other days had less effect: VEGF stimulation from d(0) stimulated less EC appearance, while starting VEGF treatment between d(3–5) resulted in a plateau in EC induction efficiency and yield (S1b Fig). Next, we checked the effects of cAMP. We added 8-bromo-cAMP at various concentrations (0.25–2 mM), time points (d(3–7)), and periods (1–5 days) together with VEGF (100 ng/mL, d(5–9)). Compatible with our previous results in mouse ESC studies, simultaneous stimulation of cAMP and VEGF enhanced EC appearance from mesoderm stage cells (S1c Fig). Transient stimulation (d(4–6)) with 1 mM 8bromo-cAMP (arrows in S1c Fig) showed the most effective outcome both in efficiency (53.6%) and yield (16.1×10^4^ cells/cm^2^), suggesting that cAMP acts on EC commitment and early EC differentiation processes, but has less effect at later stages. In our CM differentiation protocol, cells are covered with Matrigel (Thermo Fisher Scientific) solution at d(-1) to stabilize cell attachment[22]. Because cells became unstable after Activin-A treatment and massive cell detachment often occurred during EC differentiation that caused the termination of experiments, we added a secondary Matrigel overlay at d(1). This process successfully prevented massive detachment and loss of cells during EC differentiation and resulted in high EC yield. Small modification in the Activin-A treatment (125 ng/mL, 18 hours treatment) further improved cell attachment and EC appearance (data not shown). We adjusted the starting day of VEGF treatment to d(4) (the same date as cAMP treatment) to simplify the protocol. Following these modifications, we established the first endothelial differentiation protocol called “stimulation method” (Fig 1a). In summary, single dissociated hiPSCs are plated on Matrigel-coated dishes at d(-4) (60,000 to 87,500 cells/cm^2^; input hiPSCs) and cultured in the maintenance condition. The first Matrigel overlay is on d(-1), and on d(0), the medium is changed to differentiation medium (RPMI1650 with B27 supplement). Then, Activin-A (125 ng/mL) is added for 18 hours followed by medium change with BMP4 (10 ng/mL) and bFGF (10 ng/mL) treatment and the second Matrigel overlay. VEGF (100 ng/mL; d4-9) and 8bromo-cAMP (1.0 mM; d(4–6)) treatments start on d(4). EC appearance is evaluated on d(9).

### Evaluation of the stimulation method

Next, we evaluated the stimulation method by measuring EC differentiation. The addition of both VEGF and 8bromo-cAMP were compared to those with VEGF alone or with neither VEGF nor 8bromo-cAMP (vehicle). The efficiency of EC induction was evaluated with flow cytometry on d(9). The VE-cadherin^+^ EC population was significantly increased with the addition of VEGF and 8bromo-cAMP compared to VEGF alone or vehicle (56.2±12.5% vs. 11.8±7.2% vs. 2.3±2.4% of total cells, P = 0.000017, n = 4) (Fig 1b). The calculated EC count was also notably increased following the VEGF and 8bromo-cAMP treatment (1.66±0.70×10^5^ vs. 4.9±3.3×10^4^ vs. 9.8±10.4×10^3^ cells/cm^2^ culture surface, P = 0.0022, n = 4) (Fig 1c). On the other hand, the cardiac troponin T^+^ CM population decreased with VEGF and 8bromo-cAMP treatment (20.0±13.2% vs. 47.6±25.7% vs. 55.9±12.1% of total cells, P = 0.049, n = 4), while the populations of Tra-1-60^+^ undifferentiated hiPSCs and platelet-derived growth factor receptor β (PDGFRβ)-positive vascular mural cells were unchanged (S2 Fig). Overall, the cell populations on d(9) using the above protocol consisted of 60% VE-Cadherin^+^ ECs, 20% cTnT^+^ CMs, 5% PDGFRβ^+^ vascular mural cells, and 10% TRA1-60^+^ undifferentiated hiPSCs. Induced ECs (up to 70%) were purified by FACS or MACS using anti-VE-cadherin and/or CD31 antibodies, which provided ECs at more than 99% purity (Fig 1d).

**Fig 2:**
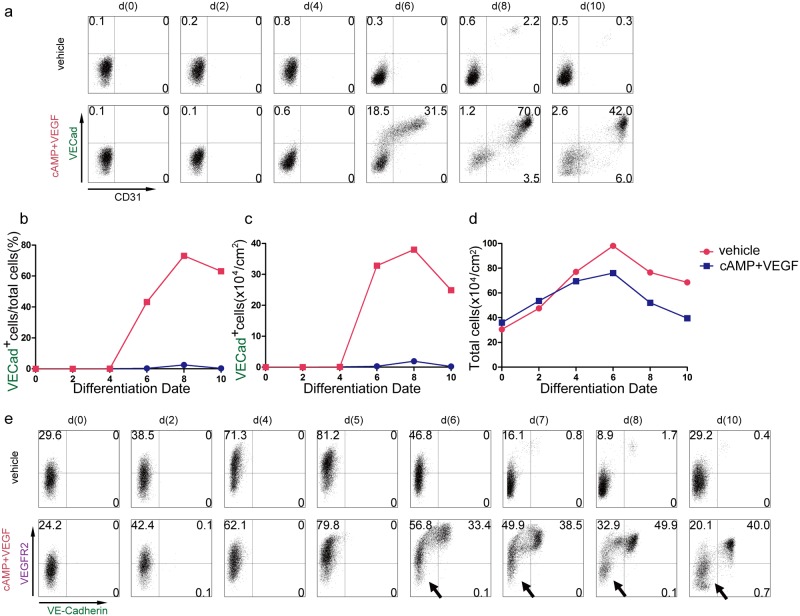
Time course of endothelial cell and pre-endothelial cell marker. (a) Representative expression time course of VE-cadherin (VECad) and CD31 under stimulation method (VEGF+cAMP) or vehicle without VEGF and cAMP by FACS. (b) Time course of VE-Cadherin-positive cell ratio in two groups. (c) Yield of VE-Cadherin positive endothelial cells per 1cm^2^ in two groups. (d) Time course of total cell counts in two groups. (e) Representative expression time course of VEGF receptor 2 (VEGFR2) and VE-cadherin in stimulation method (VEGF+cAMP) or vehicle without cAMP and VEGF. Arrows: non-responder cells to VEGF and cAMP stimulation.

Next, we examined the time course of cell differentiation. Appearance of Tra-1-60 (undifferentiated hiPSCs), VEGFR2 (mesoderm and ECs), PDGFRα (mesoderm) VE-cadherin (ECs), CD31 (ECs), PDGFRβ (mesoderm and mural cells), and VCAM1 (CMs) were examined by FACS from d(0) to d(10) with or without VEGF and 8bromo-cAMP. At d(2), almost all cells were TRA-1-60^+^ undifferentiated iPSCs (S3 Fig). At d4, the number of TRA-1-60^+^ cells decreased and the mesoderm population (VEGFR2^+^, PDGFRα^+^, and PDGFRβ^+^) started to appear. After the addition of VEGF and 8bromo-cAMP (d(4)), cell populations dramatically changed (Fig 2a–2c). From d(6) to d(8), almost no ECs (VE-cadherin^+^/CD31^+^) appeared in the vehicle condition (Fig 2a). Furthermore, VEGFR2 expression was decreased, PDGFR expressions were sustained and VCAM1^+^ CMs were first observed from d(8) (S3 Fig). On the other hand, in the VEGF and 8bromo-cAMP treatment group, ECs started to appear from d(6) and became prominent on d(10) (Fig 2a). The VEGFR2^+^ population was maintained, but the PDGFR^+^ populations gradually disappeared and almost no CMs (VCAM1^+^) were observed. After d(8), VE-cadherin^+^/CD31^+^ cells made the majority of the cell population (Fig 2a). Furthermore, the percentage (70%) and total number of VE-cadherin^+^/CD31^+^ cells peaked on d(8) (Figs 1d, 2b and 2c). However, total cell number peaked on d(6) and decreased thereafter (Fig 2d). Thus, the stimulation method, which uses VEGF and cAMP stimulation, successfully induced the EC fate from the mesoderm cells.

Despite the good effects of VEGF and cAMP on EC commitment, we noticed that not all mesoderm cells responded and committed to EC lineage when we examined the time course of VEGFR2^+^ mesoderm population (Fig 2e). VEGFR2^+^ mesoderm cells were induced by Activin-A/BMP4/bFGF treatment during d(0–4). In the control, VEGFR2^+^ cell population reached maximum on d(5) and then decreased with almost no VE-cadherin^+^ EC appearance. On the other hand, VEGF and 8bromo-cAMP treatment induced VE-cadherin^+^ ECs that maintained VEGFR2 expression after d(6). While many cells started to express VE-cadherin on d(6), a small population of cells remained negative for VEGFR2. These VEGFR2-negative cells never disappeared even after EC differentiation (d(10))(arrows in Fig 2e), indicating that they did not respond to EC commitment signaling. We speculated that these “non-responder cells” should be a main cause of the contamination of non-ECs after EC differentiation with the stimulation method.

### Second EC differentiation protocol: Stimulation-elimination method

**Fig 3:**
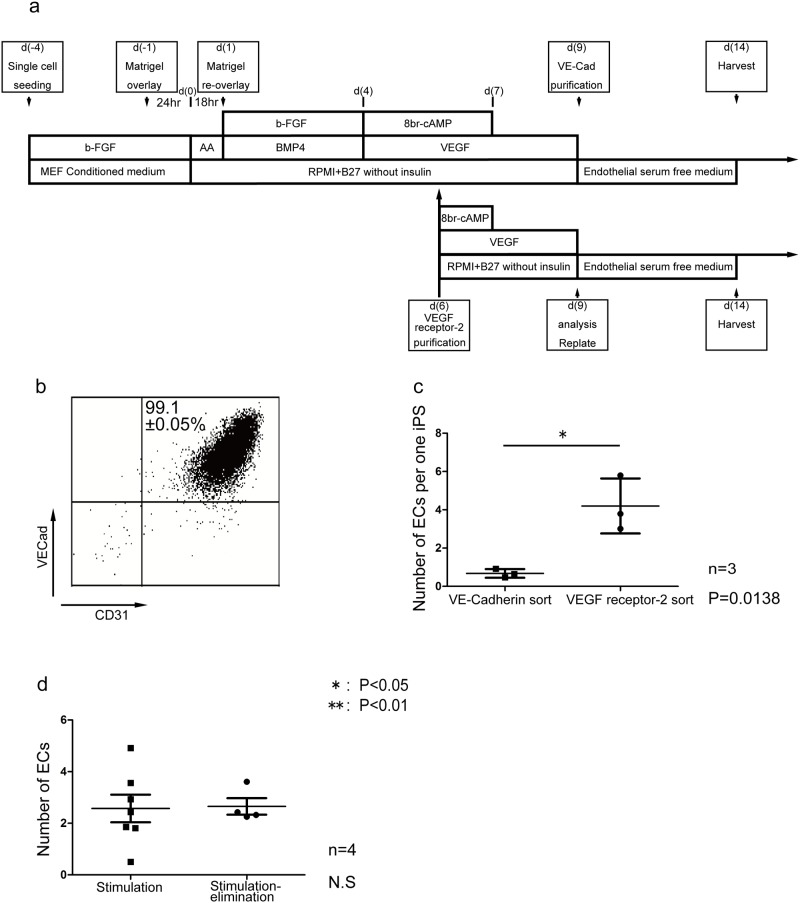
Stimulation-elimination method for efficient endothelial cells induction from human pluripotent stem cells. (a) Schematic representation of stimulation-elimination method. (b) Distinct expression pattern of VE-cadherin (VECad) and CD31 in stimulation-elimination method at differentiation day 9. (c) The yield of endothelial cells at differentiation day 9 from one human pluripotent stem cell in stimulation method or stimulation-elimination method. (d) The yield of endothelial cells at differentiation day14 from one replated endothelial cells at differentiation day 9 in two groups.

To further improve the EC induction efficiency, we therefore examined the elimination of these non-responder cells at an early EC differentiation stage. We purified VEGFR2^+^ cells on d6 with 99.0±0.5% purity (n = 7) and re-cultured them with VEGF and 8bromo-cAMP (Fig 3a). 8bromo-cAMP was withdrawn within one day (d(7)). On d(9), the responder cells gave rise to ECs with more than 99% purity (Fig 3b). Moreover, the yield of ECs increased by a factor of four compared to the stimulation method, which did not include the elimination (0.68±0.13 ECs vs. 4.20±0.83 ECs from 1 input hiPSC, n = 3) (Fig 3c). These results indicate that the selection and re-culture of VEGFR2^+^ cells on d(6) successfully eliminates non-responder cells and achieves almost complete EC induction from the responders. This second method, which combines VEGF and cAMP stimulation with non-responder elimination (“stimulation-elimination” method), showed highly specific and efficient EC differentiation. Purified ECs on d(9) induced with the stimulation-elimination method or stimulation method were similarly expanded approximately 2.5 times after an additional 5-day culture in endothelial serum free medium (Human Endothelial-SFM) (Fig 3d). This additional culture resulted in 11.1 ECs and 1.74 ECs per 1 input hiPSC on d(14) using the stimulation-elimination method and stimulation method, respectively. ECs were replated at 10,000 cells/cm^2^ following dissociation with Accumax (Innovative Cell Technologies) and grew to 80–90% confluence within 5 to 7 days of culture. In many cases, ECs showed a tendency to cease growing following an additional one to three passages in the human endothelial serum free medium condition. Occasionally, ECs were able to continuously proliferated reaching approximately 10^3^−10^4^ times increase in 2 months of culture period. Induced ECs were cryopreserved using a freeze-preserving liquid (Cellbanker III; Nippon Zenyaku Kogyo Co, Koriyama, Japan) on both d(9) and d(14). Approximately two thirds of the plated cells survived the day after thawing and grew healthily afterwards.

### Robust differentiation in multiple hiPSC lines

We confirmed robust effectiveness of our differentiation methods in two other hiPSC lines, 201B7 and 836B3 cells (S4 Fig). In the stimulation method, addition of VEGF and 8bromo-cAMP from the mesoderm stage (d(4)) significantly increased VE-cadherin^+^ EC percentages compared with percentages in the VEGF only and vehicle groups on d9 (836B3, 51.2±8.5% vs. 28.3±6.9% vs. 4.7±3.9% of total cells, P = 0.0005, n = 3; and 201B7, 44.4±14.6%, 15.7±11.1%, 5.4±0.9% of total cells, P = 0.0100, n = 3), as well as the calculated EC counts (836B3, 2.79±0.27×10^5^ vs. 1.82±0.29×10^5^ vs. 3.5±3.0×10^4^ cells/cm^2^ culture surface, P = 0.0001, n = 3; and 201B7, 1.89±0.44×10^5^ vs. 8.4±7.4×10^4^ vs. 2.3±0.3×10^4^ cells/cm^2^ culture surface, P = 0.0175, n = 3). Similarly, the stimulation-elimination method was effective in multiple cell lines. After purification and re-culturing of VEGFR2^+^ cells on d6, more than 99% efficiency of VE-cadherin^+^/CD31^+^ cells was achieved on d(9) from all cell lines examined. The elimination of non-responder cells noticeably increased the yield of ECs compared between the stimulation-elimination and stimulation methods (836B3, 2.26±0.59 vs. 0.70±0.28 ECs from 1 hiPSC input, P = 0.076, n = 3; and 201B7, 3.42±0.42 vs. 0.69±0.11 ECs from 1 hiPSC input, P = 0.0046, n = 3). Thus, our methods were robust and effective in multiple hiPSC lines.

### Characterization of hiPSC-derived ECs

**Fig 4:**
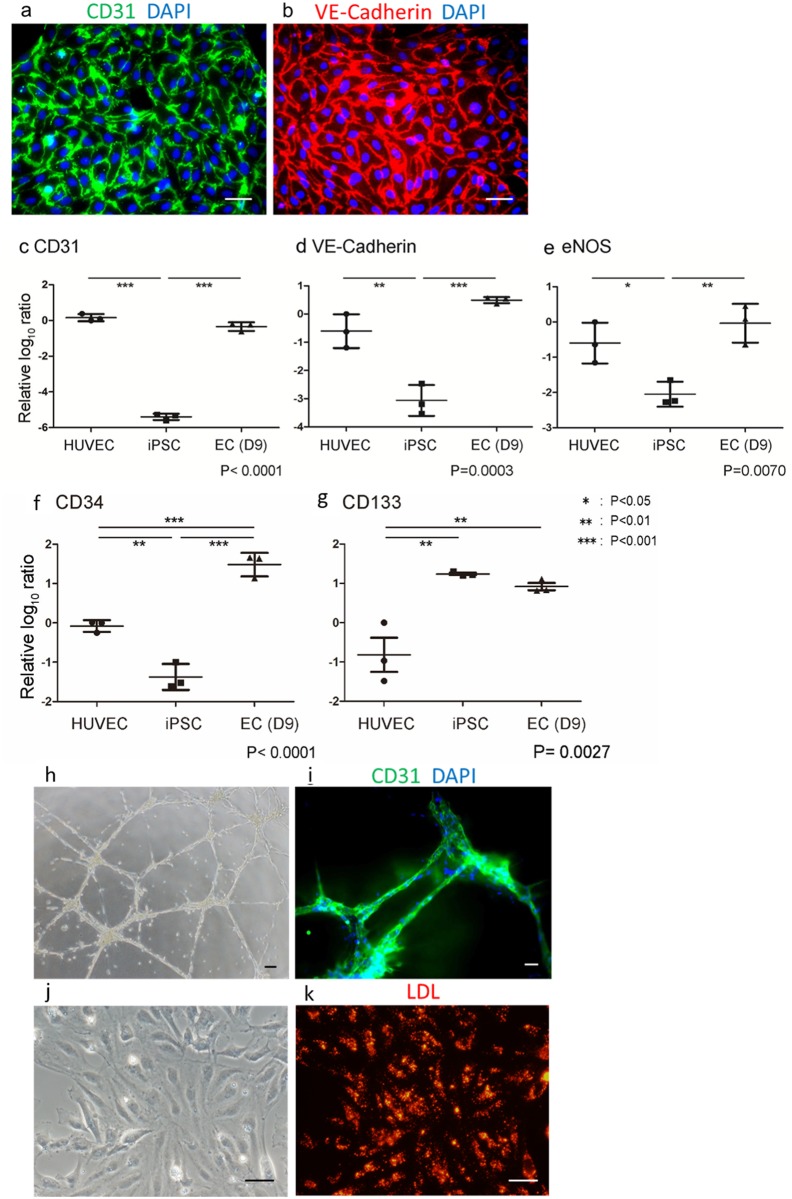
Characterization of endothelial cell induced from human iPS cell with stimulation-elimination method. Immunofluorescent staining for (a) CD31 (green) and (b) VE-Cadherin (red) in human iPS cell-derived endothelial cells. Nuclei were visualized with DAPI (blue). (c-g) Relative mRNA log_0_ ratio in endothelial cells at differentiation day 9 (EC (D9)) and undifferentiated human iPS cell (iPSC) compared with human umbilical vein endothelial cell (HUVEC). CD31 (c), VE-Cadherin (d), eNOS (e), CD34 (f), and CD133 (g). (h) Tube formation assay. Human iPS cell-derived endothelial cells were recultured on Matrigel Basement Membrane Matrix GFR coated dish. (i) Immunofluorescent stained of CD31 for recultured cells on Matrigel. (j,k) Acetyl-LDL incorporation assay. Endothelial cells were incubated with acetylated LDL labeled with 1,1’-dioctadecyl-3,3,3’,3’-tetramethylindo-carbocyanine perchlorate (DiI-Ac-LDL). Bright-field (j) and fluorescent (k) images. Scale bars: 50 μm in (a), (b) and (i), 100 μm in (h), 200 μm in (j) and (k).

hiPSC (201B6)-derived pure ECs were cultured on a gratin coated dish and positively immunostained for the endothelial markers CD31 and VE-cadherin (Fig 4a and 4b). The mRNA expression levels of the endothelial markers CD31, VE-cadherin and endothelial nitric oxide synthase (eNOS) in hiPSC-derived ECs on d(9) were comparable to those in HUVECs (human umbilical vein endothelial cells) and significantly higher than those in undifferentiated hiPSCs (negative control)(Fig 4c–4e). CD34[28] and CD133[29] are markers of ECs as well as multipotent progenitor cells, including immature hematopoietic stem cells and precursors of endothelial cells[30,31]. CD34 and CD133 expression levels of hiPSC-derived ECs were higher than those of HUVECs (Fig 4f and 4g). Though the expression levels had decreased during the culturing of ECs, they were still higher than in HUVEC, even on d(30) (data not shown). These results suggest that our methods achieve induction and maintenance of an early stage ECs. We next tested tube formation assay. ECs (d(13)) successfully formed tube-like networks on Matrigel (Fig 4h). These cells configuring tube formation were positive for CD31 (Fig 4i). Uptake of low-density lipoproteins (LDLs), another EC function, was clearly displayed in hiPSC-derived ECs (Fig 4j and 4k). The tube formation capacity and morphology were almost comparable with those of HUVECs (S5 Fig). Those results suggest that hiPSC-derived ECs with stimulation-elimination method are functional ECs.

### Arterial and venous specification of hiPSC-derived EC

Previously, we reported that cAMP signaling is involved in arterial endothelial specification through Notch and β-catenin activation in a mouse ES cell system[21]. Notch signal related-genes, including Notch1 and 4, Dll4, RBP-J, and Hey1/Hey2, are essential for arterial formation and vasculature development[32–36]. On the other hand, chicken ovalbumin upstream promoter-transcription factor II (COUP-TFII) suppresses Notch signaling and regulates vein identity[37]. We recently demonstrated that angiopoietin 1 induces venous ECs in coronary vein formation in vivo and EC differentiation from mouse embryonic stem cells[38].

**Fig 5:**
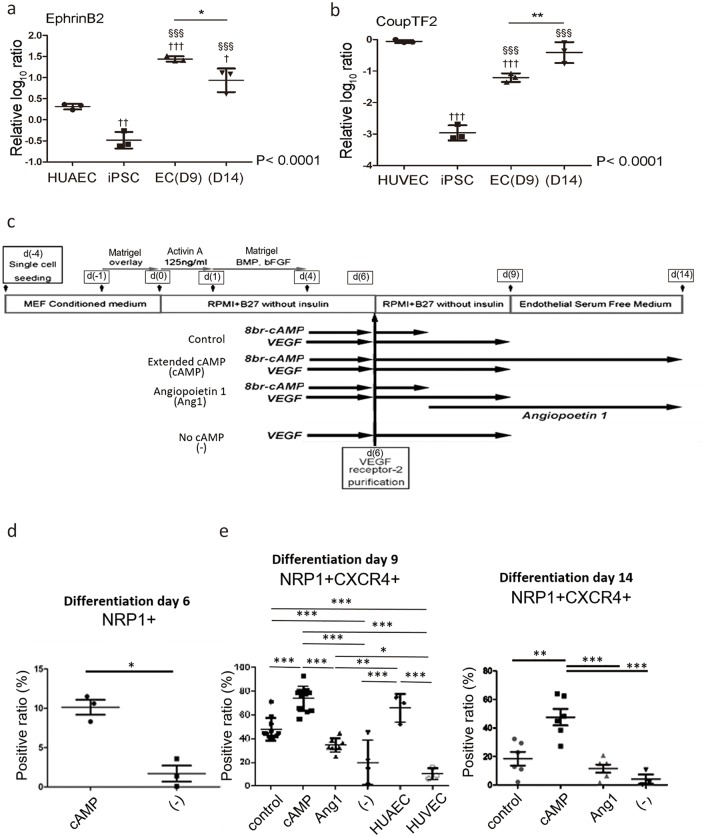
Arterial and venous specification of endothelial cells derived from human induced pluripotent stem cells with supplementation of cAMP or angiopoetin1. (a,b) mRNA expression of arterial and venous endothelial cell maker in endothelial cells derived from human iPS cell with stimulation-elimination method. Relative mRNA log_10_ ratio of EphrinB2 (a) and CoupTF2 (b) in endothelial cell induced from human iPS cell at differentiation day 9 (EC D9), at day14 (D14), human iPSC and umbilical endothelial cells compared with HUVEC. †, significant difference compared with (a) HUAEC or (b) HUVEC. §, significant difference compared with human iPS cell. (c) Schematic representation of the arterial and venous specification methods. (d) Mean ratios of NRP1-positive cells in stimulation-elimination method groups (control) to no cAMP group ((-)) at differentiation day 6. (e) Mean ratios of NRP1- CXCR4-double positive cells at differentiation day 9 and 14 in control, extended cAMP groups (cAMP), angiopoetin1 groups (Ang1), no cAMP groups (-), and HUAECs / HUVECs (day 9 only). *P<0.05, **P<0.01, ***P<0.001.

Then, we examined arterial-venous specification in human ECs induced from iPSCs. As being speculated from that ECs are induced with VEGF and cAMP stimulation, ECs that induced with our method were found to be initially induced with arterial features that show 13.5 times more mRNA expression for the arterial EC marker ephrinB2[39] than do human umbilical artery endothelial cells (HUAECs) on d(9). However, following an additional 5-day culture for EC expansion with no cAMP, ephrinB2 expression (d(14)) significantly decreased but still higher than that in HUAECs (Fig 5a). Other arterial marker expressions, such as Dll1, Dll4, and Notch1, were similarly high in ECs on d(9) and showed a tendency to decrease by d(14) (S6 Fig). On the other hand, whereas the mRNA expression of a venous EC marker, COUP-TFII[37] on d(9) was significantly lower than that in HUVECs, it returned to a comparable level after EC expansion (Fig 5b). We previously showed that arterial-venous specification was unstable in the early stage of ECs in a mouse ESC system[21]. Early ECs still possess plasticity between arterial and venous phenotypes, and they can change their features from arterial to venous and vice versa according to the culture conditions. Therefore, we speculated that the ECs induced from hiPSCs in this study remained in an undetermined state for arterial and venous ECs. Higher expressions of early stage EC markers CD34 and CD133 (Fig 4f and 4g) further support our speculation that ECs retain plasticity in EC diversity.

Next, we tried to control arterial and venous specification with various combinations of small molecules or growth factors in our stimulation-elimination method (Fig 5c–5e). The ratio of NRP1-positive cells at d6 in stimulation-elimination method or NRP1 and CXCR4 double positive ECs at d(9) were significantly increased compared to those with VEGF alone (no cAMP) condition. At d(14), ECs with VEGF alone condition completely lost arterial maker. Moreover, extended cAMP supplementation after d(7) had beneficial effect for arterial specification at d(9) and d(14). The positive ratio of NRP1 and CXCR4 at d(9) was almost comparable with those in HUAECs (human umbilical artery endothelial cells) (Fig 5e). On the other hand, Supplementation of angiopoietin 1 after d(6) together with cAMP administration during d(4) to d(6) significantly decreased the ratio of arterial EC population at d(9). Thus, arterial-venous EC fates were able to be controlled during and after EC differentiation.

## Discussion

In this study, we describe efficient and scalable EC differentiation methods based on a 2D monolayer, serum-free culture system. Potent induction of EC commitment and differentiation with cAMP and VEGF at the mesoderm stage dramatically shifted the fate of cells toward ECs and achieved highly efficient EC induction. Moreover, the elimination of cells that had not responded to EC commitment signals (non-responder cells) at the peri-EC stage (d(6)) resulted in almost 100% differentiation efficiency to ECs. The combination of differentiation stage-specific signal stimulation and non-responder elimination (stimulation-elimination method) is a highly efficient and scalable strategy for the pure induction of target cell populations.

To generate a large-sized organ or tissue, blood vessels that are mainly formed by ECs are necessary. For example, cardiac regeneration in humans after myocardial infarction is estimated to require 10^8^ to 10^9^ cells[23,40–43]. Given that the percentage of the EC population to total cells in an adult mouse heart is around 7%, 10^7^ to 10^8^ ECs would be required to realize human heart regeneration by cell transplantation. Our method is calculated to be amenable to prepare 10^8^ ECs from just two 6-well plates robustly from several hiPSC lines, suggesting the possibility to scale the delivery of ECs even at an industrial level.

ECs are key to 3D tissue engineering, because blood vessels are necessary for mature and functional tissues and organs. ECs in vivo have been reported to have diverse phenotypes that closely relate to the tissue and organ function, such as fenestrated ECs in bone marrow, sinusoidal ECs in liver[44], ECs in glomeruli and podocytes in kidney[45], and ECs involved in the blood-brain barrier and astrocytes in brain[46]. Previously, various methods for induction of ECs from human pluripotent stem cells were reported[47–51]. Lineage control and enhanced EC differentiation with cAMP signaling combined with VEGF is our original method[16,17]. ECs induced by VEGF and cAMP showed plasticity between arterial and venous phenotypes[21]. In addition, hiPSC-derived ECs induced with our method showed expression of immature EC markers (Fig 4f and 4g), suggesting that these ECs should possess a higher ability to adjust to tissue-specific environments and diversify with appropriate function than do fully differentiated EC sources such as HUVECs. Thus, our hiPSC-derived ECs should be more suitable for the reconstitution of organ functions in vitro and in vivo. These results suggest that our stimulation-elimination method, which efficiently and robustly differentiates hiPSCs into ECs, is a potential technological basis for regenerative medicine and tissue engineering.

## Supporting information

S1 Fig Optimization of VEGF and cAMP supplementation. (a) Ratio of VE-Cadherin positive endothelial cells per total cells at differentiation day 9 with sustained addition of 0, 50 or 100 ng/ml VEGF (left) at differentiation day 3 to 9. Mean yield of endothelial cells per 1 cm2 in administration of each VEGF concentrations (right). (b) Ratio of endothelial cell per total cells at differentiation day 9 with addition of 100 ng/ml VEGF from differentiation day 0–9 (Day0), 2–9 (Day2), 3–9 (Day3), 5–9 (Day5) or no administration of VEGF ((-)) together with 1.0 mM cAMP from differentiation day 5–6 (left). Mean yield of endothelial cells per 1cm2 in each additional timing of VEGF or no administration of VEGF (right). (c) Ratio of VE-Cadherin positive cell per total cells at differentiation day 9 by flow cytometory with addition of 100 ng/ml VEGF from differentiation day 5 to day 9 together with various timing and concentration of cAMP (Upper row). Mean yield of endothelial cells per 1cm2 in each administrated condition of cAMP (Lower row).
(PDF)

S2 Fig Ratio of cardiovascular cell and undifferentiated iPSC differentiated and induced from iPSC cell with stimulation method. Ratio of (a) cardiac troponin T (cTnT), (b) Platelet-Derived Growth Factor Receptor β (PDGFRβ) and (c) TRA-1-60 positive cell per total cells at differentiation day 9 by with stimulation method (cAMP+VEGF), only VEGF administration (VEGF) and no administration (vehicle). Mean yield of (d) cTnT-positive cardiomyocyte, (e) PDGFRβ-positive vascular mural cell, (f) TRA-1-60 undifferentiated iPSC per 1cm2 in three groups.
(PDF)

S3 Fig Representative time course of cell surface marker. Expression time course of (a) TRA-1-60 and CD31, (b) TRA-1-60 and CD31, (c) PDGF-Rβand VCAM-1 with stimulation method (cAMP+VEGF) or control without cAMP and VEGF group (vehicle).
(PDF)

S4 Fig Multi cell line confirmation of efficiency and scalability in stimulation method and stimulation-elimination method. (a)(c) Ratio of VE-Cadherin-positive endothelial cells per total cells at differentiation day 9 by flow cytometry with stimulation method (cAMP+VEGF), only VEGF administration groups (VEGF) and no administration groups (vehicle) in other two iPS cell lines (836B3, 207B7). (b)(d) Yield of endothelial cells per 1cm2 in two groups. (e)(f) The yield of endothelial cells at differentiation day 9 from one hiPSC in stimulation method or stimulation-elimination method.
(PDF)

S5 Fig Tube formation assay and Acetyl-LDL incorporation assay in HUVECs. HUVECs were recultured on Matrigel Basement Membrane Matrix GFR- coated dish (left upper). Immunofluorescent stained of CD31 for recultured cells on Matrigel (right upper). Endothelial cells were incubated with acetylated LDL labeled with 1,1’-dioctadecyl-3,3,3’,3’-tetramethylindo-carbocyanine perchlorate (DiI-Ac-LDL) (lower). Bright-field (left) and fluorescent (right) images. HUVEC, human umbilical vein endothelial cells. Scar bars: 200 μm.
(PDF)

S6 Fig Relative expression of arterial markers in endothelial cells induced from human iPSC with stimulation-elimination method. mRNA log_10_ ratio of Dll1 (a), Dll4 (b) and Notch1 (c) at differentiation day 0 (D0), day 4 (D4), day 9 (D9) and day 14 (D14) compared with human umbilical vein endothelial cell (HUVEC).
(PDF)

S1 Table Fluorescence-conjugated monoclonal antibodies used for Immunofluorescence Assay (IF) and FACS analysis. (PDF)

S2 Table List of forward and reverse primer sequences for reverse transcription-polymerase chain reaction. (PDF)
