# Correction: Efficient and robust differentiation of endothelial cells from human induced pluripotent stem cells via lineage control with VEGF and cyclic AMP

**DOI:** 10.1371/journal.pone.0176238

**Published:** 2017-04-17

**Authors:** Takeshi Ikuno, Hidetoshi Masumoto, Kohei Yamamizu, Miki Yoshioka, Kenji Minakata, Tadashi Ikeda, Ryuzo Sakata, Jun K. Yamashita

The lines labeled “vehicle” and “cAMP+VEGF” are incorrectly switched in Fig 2b and 2c. Please see the corrected Fig 2 here.

**Fig 2:**
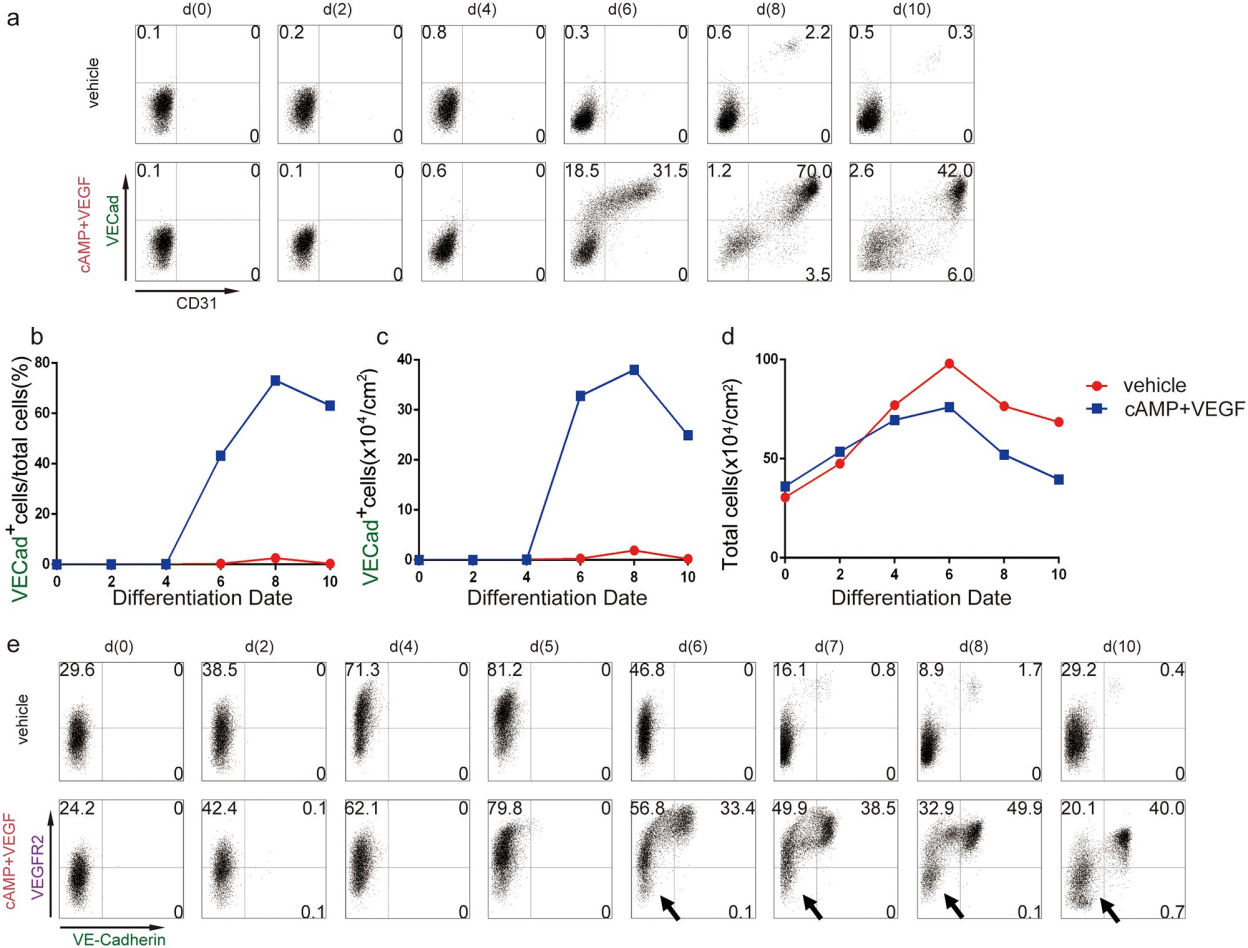
Time course of endothelial cell and pre-endothelial cell marker. (a) Representative expression time course of VE-cadherin (VECad) and CD31 under stimulation method (VEGF+cAMP) or vehicle without VEGF and cAMP by FACS. (b) Time course of VE-Cadherin-positive cell ratio in two groups. (c) Yield of VE-Cadherin positive endothelial cells per 1cm^2^ in two groups. (d) Time course of total cell counts in two groups. (e) Representative expression time course of VEGF receptor 2 (VEGFR2) and VE-cadherin in stimulation method (VEGF+cAMP) or vehicle without cAMP and VEGF. Arrows: non-responder cells to VEGF and cAMP stimulation.
