# Supplementary figures and images for: Efficient and robust differentiation of endothelial cells from human induced pluripotent stem cells via lineage control with VEGF and cyclic AMP
Source: PLoS One. 2017 Mar 13;12(3):e0173271. doi: 10.1371/journal.pone.0173271 (PMC5347991; doi:10.1371/journal.pone.0173271)

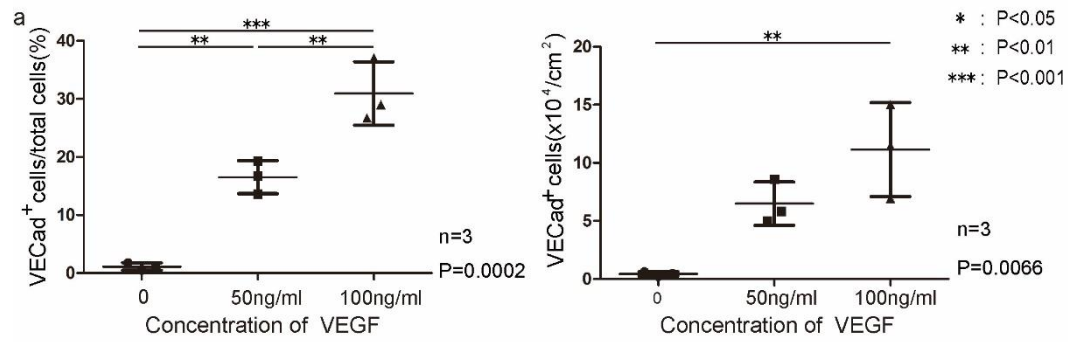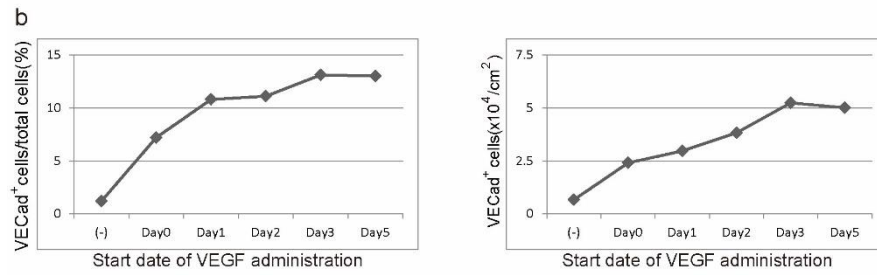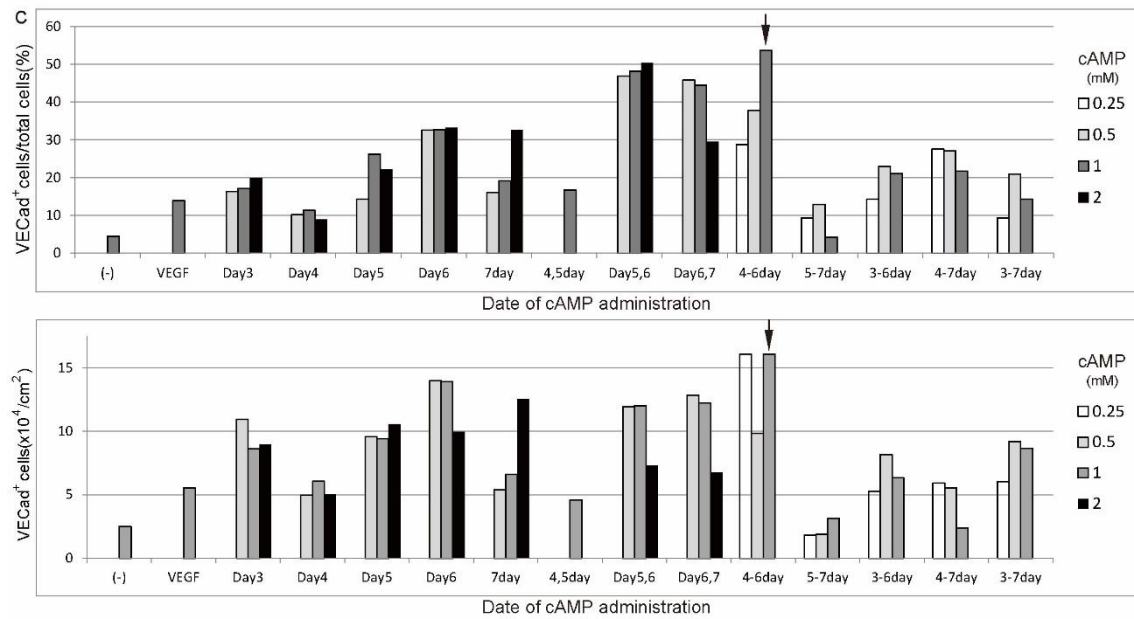

Supplement: S1 Fig — (a) Ratio of VE-Cadherin positive endothelial cells per total cells at differentiation day 9 with sustained addition of 0, 50 or 100 ng/ml VEGF (left) at differentiation day 3 to 9. Mean yield of endothelial cells per 1 cm2 in administration of each VEGF concentrations (right). (b) Ratio of endothelial cell per total cells at differentiation day 9 with addition of 100 ng/ml VEGF from differentiation day 0–9 (Day0), 2–9 (Day2), 3–9 (Day3), 5–9 (Day5) or no administration of VEGF ((-)) together with 1.0 mM cAMP from differentiation day 5–6 (left). Mean yield of endothelial cells per 1cm2 in each additional timing of VEGF or no administration of VEGF (right). (c) Ratio of VE-Cadherin positive cell per total cells at differentiation day 9 by flow cytometory with addition of 100 ng/ml VEGF from differentiation day 5 to day 9 together with various timing and concentration of cAMP (Upper row). Mean yield of endothelial cells per 1cm2 in each administrated condition of cAMP (Lower row). (PDF) [file pone.0173271.s001.pdf]

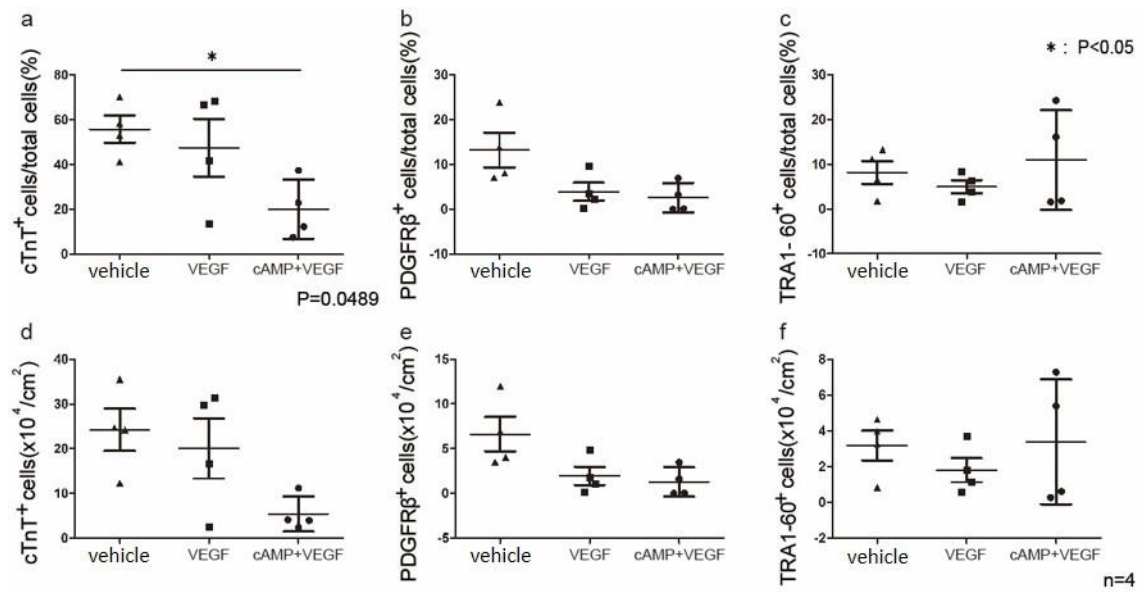

Supplement: S2 Fig — Ratio of (a) cardiac troponin T (cTnT), (b) Platelet-Derived Growth Factor Receptor β (PDGFRβ) and (c) TRA-1-60 positive cell per total cells at differentiation day 9 by with stimulation method (cAMP+VEGF), only VEGF administration (VEGF) and no administration (vehicle). Mean yield of (d) cTnT-positive cardiomyocyte, (e) PDGFRβ-positive vascular mural cell, (f) TRA-1-60 undifferentiated iPSC per 1cm2 in three groups. (PDF) [file pone.0173271.s002.pdf]

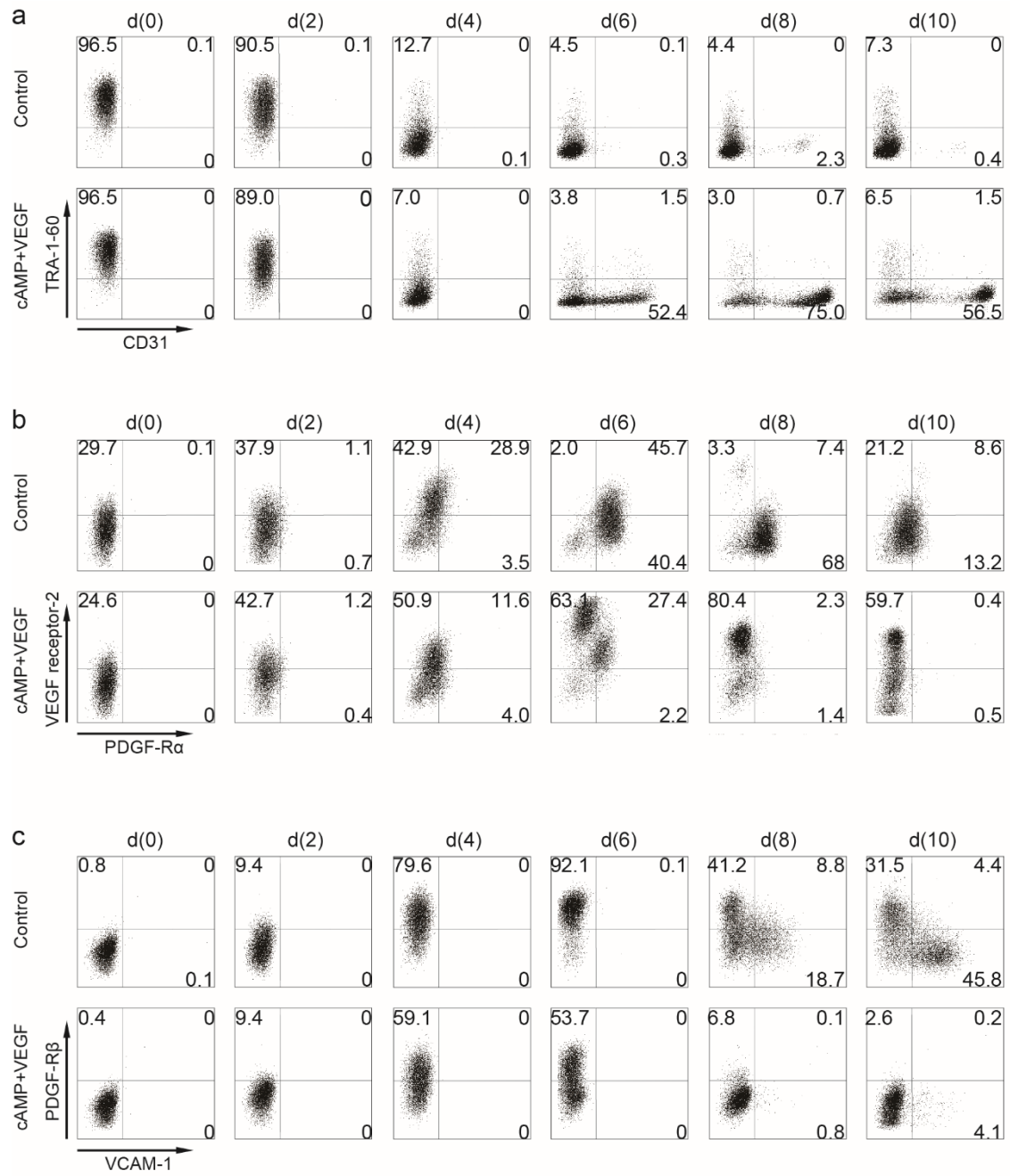

Supplement: S3 Fig — Expression time course of (a) TRA-1-60 and CD31, (b) TRA-1-60 and CD31, (c) PDGF-Rβand VCAM-1 with stimulation method (cAMP+VEGF) or control without cAMP and VEGF group (vehicle). (PDF) [file pone.0173271.s003.pdf]

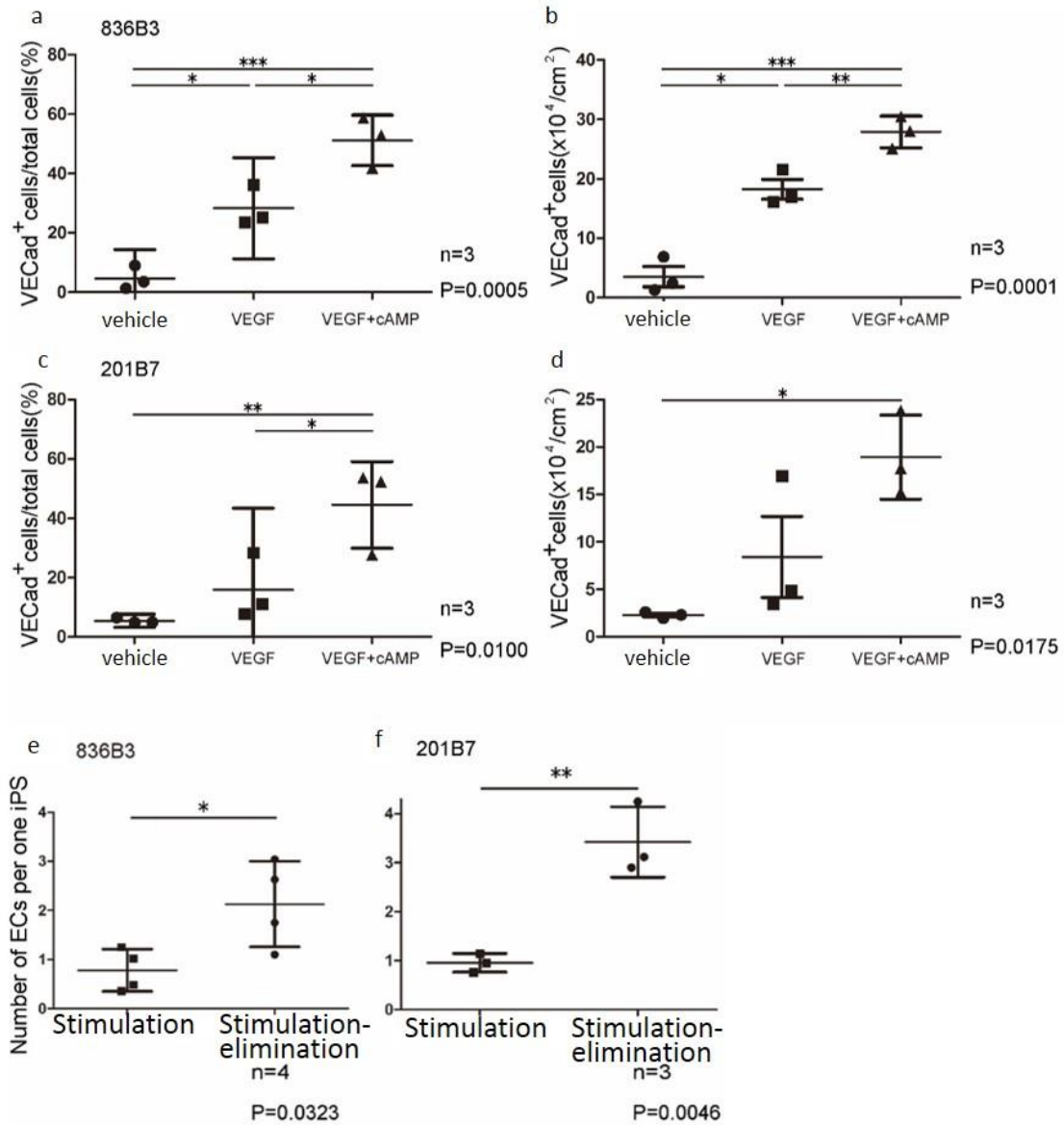

Supplement: S4 Fig — (a)(c) Ratio of VE-Cadherin-positive endothelial cells per total cells at differentiation day 9 by flow cytometry with stimulation method (cAMP+VEGF), only VEGF administration groups (VEGF) and no administration groups (vehicle) in other two iPS cell lines (836B3, 207B7). (b)(d) Yield of endothelial cells per 1cm2 in two groups. (e)(f) The yield of endothelial cells at differentiation day 9 from one hiPSC in stimulation method or stimulation-elimination method. (PDF) [file pone.0173271.s004.pdf]

CD31 DAPI

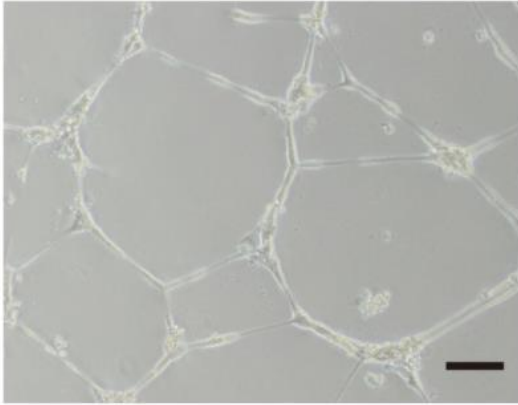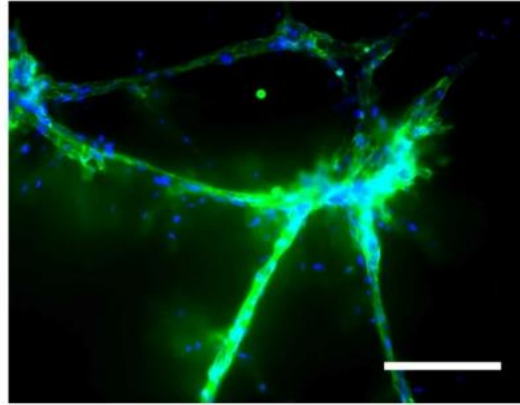

LDL

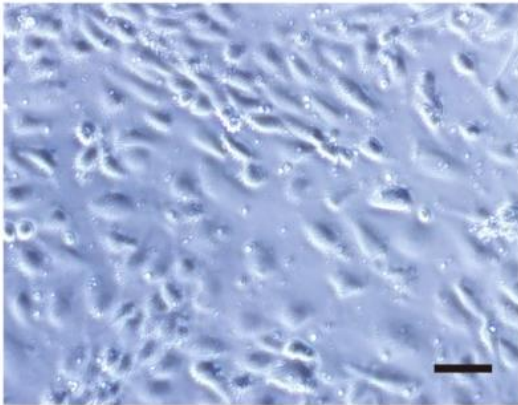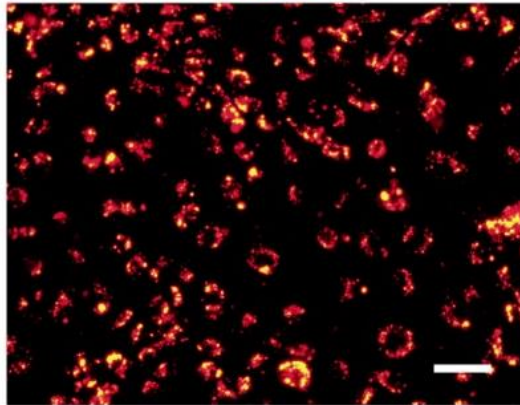

Supplement: S5 Fig — HUVECs were recultured on Matrigel Basement Membrane Matrix GFR- coated dish (left upper). Immunofluorescent stained of CD31 for recultured cells on Matrigel (right upper). Endothelial cells were incubated with acetylated LDL labeled with 1,1’-dioctadecyl-3,3,3’,3’-tetramethylindo-carbocyanine perchlorate (DiI-Ac-LDL) (lower). Bright-field (left) and fluorescent (right) images. HUVEC, human umbilical vein endothelial cells. Scar bars: 200 μm. (PDF) [file pone.0173271.s005.pdf]

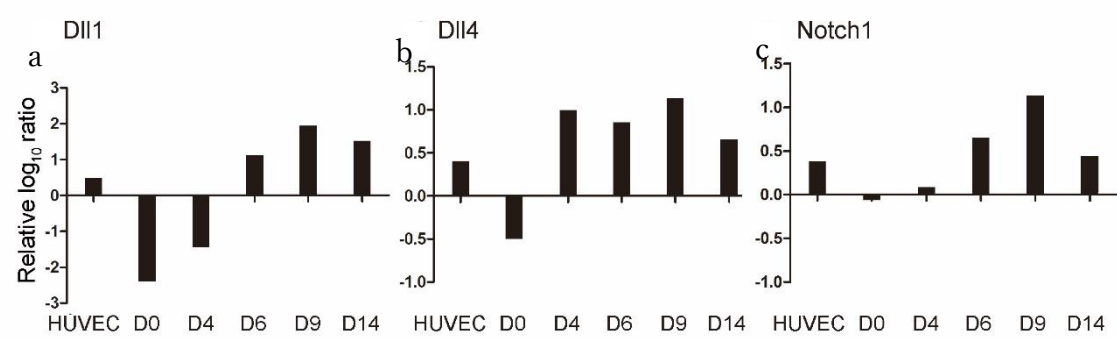

Supplement: S6 Fig — mRNA log10 ratio of Dll1 (a), Dll4 (b) and Notch1 (c) at differentiation day 0 (D0), day 4 (D4), day 9 (D9) and day 14 (D14) compared with human umbilical vein endothelial cell (HUVEC). (PDF) [file pone.0173271.s006.pdf]
