# Supplementary material for: Efficient and robust differentiation of endothelial cells from human induced pluripotent stem cells via lineage control with VEGF and cyclic AMP
Source: PLoS One. 2017 Mar 13;12(3):e0173271. doi: 10.1371/journal.pone.0173271 (PMC5347991; doi:10.1371/journal.pone.0173271)
Supplement: S1 Table — (PDF) [file pone.0173271.s007.pdf]

Antibody	Clone	Host	Conjugated fluorescence		Supplier
CD31	390	Mouse	APC	FACS	eBioscience
VE-cadherin	55-7H1	Mouse	PE	FACS	BD Bioscience
VE-cadherin	55-7H1	Mouse	FITC	FACS	BD Bioscience
VEGER2/KDR	ES8-2-E6	Mouse	PE	FACS	Miltenyi Biotech
PDGFRa	PRa292	Mouse	APC	FACS	R&D
PDGFRb	28D4	Mouse	PE	FACS	BD Bioscience
VCAM1	STA	Mouse	APC	FACS	Biolegend
TRA-1-60	TRA-1-60	Mouse	FITC	FACS	BD Bioscience
CD31	9G11	Mouse	Alexa Fluor 488	IF	R&D
VE-cadherin	16B1	Mouse	PE	IF	eBioscience
Claudin 5	4C3C2	Mouse	Alexa Fluor 498	IF	Invitrogen
