# Supplementary material for: Efficient and robust differentiation of endothelial cells from human induced pluripotent stem cells via lineage control with VEGF and cyclic AMP
Source: PLoS One. 2017 Mar 13;12(3):e0173271. doi: 10.1371/journal.pone.0173271 (PMC5347991; doi:10.1371/journal.pone.0173271)
Supplement: S2 Table — (PDF) [file pone.0173271.s008.pdf]

Gene sequence	Sense	Antisense
GAPDH	GCACCGTCAAGGCTGAGAAC	TGGTGAAGACGCCAGTGGA
CD31	ATTGCAGTGGTTATCATCGGAGTG	CTCGTTGTTGGAGTTCAGAAGTGG
VE-Cadherin	TCGTCATGGACCGAGGTT	TCTACAATCCCTTGCAGTGTGA
eNOS	GCGGCTGCATGACATTGAG	GTCGCGGTAGAGATGGTCAAG
ephrinB2	CTCCTCAACTGTGCCAAACCA	GGTTATCCAGGCCCTCCAAA
CoupTF2	TGGTTCCAAACCAGTTTATTCTGTG	AAGTGCGTTTCCATCATCTTTGAG
Dll1	ACTGCAGCTCTTCACCCTGT	CAGGTGCAGGAGAAGTCGTT
Dll4	GCCTGGACAAGTCCAACTGT	CGCTGATATCCGACACTCTG
Notch1	CAGGCAATCCAGGACTATG	CAGGCGTGTTGTTCTCACAG
CD34	TCTGATCTCCATGGCTTCCT	ACTGAGGCAACAGCTCAACC
CD133	TCTGGGTCTACAAGGACTTTCC	TCTGGGTCTACAAGGACTTTCC
